# An ultrasound-based artificial intelligence framework for difficult airway prediction: A two-model, three-step decision framework

**DOI:** 10.1371/journal.pone.0342339

**Published:** 2026-02-18

**Authors:** Chunmeng Fu, Cunyuan Luan, Huabo Liu, Wenfei Wang, Xia Zhou, Yuanfang Jia, Bing Ding, Lei Zhang, Li Yuan, Zejun Niu

**Affiliations:** 1 Department of Anesthesiology, The Affiliated Hospital of Qingdao University, Qingdao, China; 2 School of Automation, Qingdao University, Qingdao, China; 3 Shandong Key Laboratory of Industrial Control Technology, Qingdao, China; 4 Department of Anesthesiology, Qingdao Traditional Chinese Medicine Hospital, Qingdao Hiser Hospital Affiliated of Qingdao University, Qingdao, China; 5 Department of Anesthesiology, Shandong Provincial Key Medical and Health Laboratory of Anesthesia and Brain Function, Qingdao, China; Azienda Ospedaliero Universitaria Careggi, ITALY

## Abstract

**Background:**

At present, the early warning of difficult airway remains fraught with challenges. Previous ultrasonic quantitative parameters have demonstrated favorable application potential in difficult airway assessment, and deep learning techniques have also exhibited satisfactory performance in the interpretation of this condition. Based on this, we aim to construct a “two-model, three-step” hierarchical strategy, develop an ultrasound image-based artificial intelligence (AI) framework for difficult airway prediction, and conduct its internal validation.

**Methods:**

In this study, we included 903 patients who underwent elective general anesthesia surgery at the Affiliated Hospital of Qingdao University between May 2024 and April 2025. 752 cases were used for model training and validation, and 151 cases served as an internal test set. Four planes of neck ultrasound images were scanned for each patient and used to develop two artificial intelligence models (based on convolutional neural networks): CL-AI for initial screening and VIDIAC-AI for secondary risk stratification. Model performance was evaluated using five-fold cross-validation and internal testing. External validation was not performed.

**Results:**

Among 903 patients, difficult laryngoscopy occurred in 189 cases (20.9%) under direct laryngoscopy and in 50 cases (5.5%) under video laryngoscopy. In the independent test set, the CL-AI model achieved an AUC of 0.86 (95% CI: 0.79–0.91), with an accuracy of 0.84, sensitivity of 0.84, specificity of 0.84, precision of 0.59, and an F1 score of 0.69. The VIDIAC-AI model achieved an AUC of 0.82 (95% CI: 0.75–0.88), with an accuracy of 0.81, sensitivity of 0.75, specificity of 0.81, precision of 0.18, and an F1 score of 0.29.

**Conclusions:**

This study proposes an ultrasound-based AI framework for risk stratification of difficult laryngoscopic exposure. The Two-Model, Three-Step decision framework is intended as a clinician decision-support tool, not an independent diagnostic method, and requires further validation in large multicenter cohorts.

## Introduction

Airway management is a core component of clinical practice in the intensive care unit (ICU), emergency department, and anesthesiology department [1,2]. In routine airway management, physicians across different departments exhibit variations in laryngoscope selection; the choice between direct laryngoscopes (DL) and video laryngoscopes (VL) is highly dependent on individual physicians’ clinical experience, and no unified standard protocol has been established [3]. Additionally, the application of both devices involves practical cost considerations: the procurement and maintenance costs of VL are significantly higher than those of DL, and its operation requires physicians to receive systematic specialized training [4]. In terms of clinical efficacy, some studies have confirmed that VL can improve the degree of glottic visualization and increase the first-attempt intubation success rate in emergency scenarios [5,6]. However, other studies have noted that in critically ill patients in the emergency department or ICU, VL does not significantly improve intubation outcomes [7,8], and its effectiveness depends on the operator’s clinical experience and procedural proficiency [4]. Consequently, the development of a difficult airway is associated not only with patient-specific factors but also with the physician’s experience and the selection of intubation devices.

Airway assessment is a key step in preoperative airway risk stratification and assisting physicians in conducting airway management [9]. Traditional airway assessment methods (e.g., Mallampati classification, mouth opening, and mentohyoid distance) are highly influenced by the operator’s subjective factors, when used alone or in simple combinations, their predictive accuracy is relatively low, which makes them difficult to meet clinical needs [10,11]. Ultrasound has been widely used for preoperative assessment of difficult airways due to its convenience, non-invasiveness, real-time imaging capabilities, and ability to clearly display cervical anatomical structures (such as the hyoid bone and epiglottis) [12]. Previous studies have shown that the distance from skin to hyoid bone, epiglottis, vocal cord and other anatomical structures can be used as an ultrasound index to predict difficult airways [13–15], and has shown good predictive performance to a certain extent. However, such methods are highly dependent on manual measurement and are easily affected by operator experience and measurement plane differences, which limits their generalization ability and practical application.

In recent years, artificial intelligence (AI) models based on deep learning, especially convolutional neural networks (CNN), have been widely used in the medical field [16,17]. Regarding airway assessment, previous studies have attempted to build deep learning models based on facial image features [18–21] or develop predictive models using laryngeal sound signals [22,23], which have improved the prediction performance of difficult laryngoscopy exposure to some extent. However, most of these studies focus on predicting difficult laryngoscopy exposure under DL, with only a few studies (Xia et al. [19]) involving VL scenarios. Therefore, existing models can usually only determine whether a patient will experience difficult exposure under a single type of laryngoscopy, and it is still difficult to achieve airway risk stratification covering both DL and VL.

Based on the aforementioned shortcomings, we believe that accurate airway risk stratification relies on reliable assessment tools and predictive models, and these models should be applicable to both direct DL and VL clinical scenarios. Compared to facial images or voice signals, ultrasound images can directly reflect the deep anatomical structures of the anterior neck and airway, providing a clearer anatomical basis for airway assessment. Although ultrasound has been used for quantitative measurement of airway anatomical structures, there is currently a lack of research combining ultrasound images with deep learning methods for risk stratification or classification of difficult laryngoscopy exposure. Drawing on this, we propose the hypothesis that AI models can distinguish between difficult laryngoscopic exposure under DL and VL using ultrasound images. If a patient’s airway risk can be clearly identified, it would be possible to consult or refer to experienced physicians, select appropriate intubation devices, and thereby avoid airway-related complications caused by inappropriate selection of intubation devices.

This study aims to construct AI models using deep learning (CNN) to classify difficult visualization under DL and VL, achieve airway risk stratification for patients, and establish a correlation between ultrasound images and actual difficult laryngoscopic exposure.

## Materials and methods

**Fig 1:**
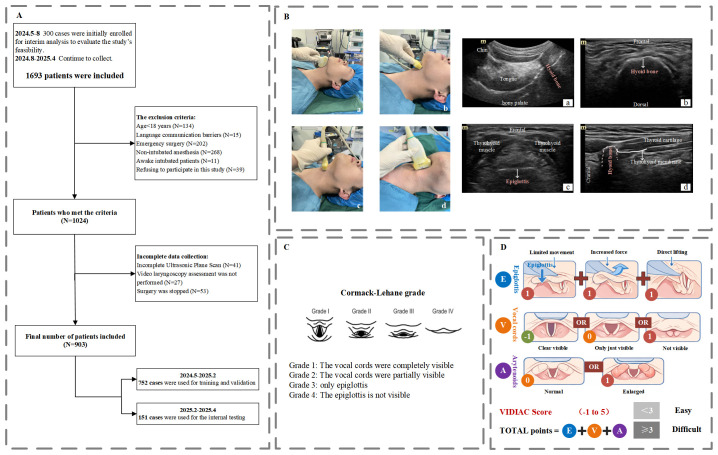
Schematic Overview of the Study. **(A) Flowchart illustrating patient inclusion/exclusion criteria and interim analysis procedures.** After enrollment of the initial 300 patients, an interim analysis was performed to evaluate preliminary model performance. Due to insufficient generalizability observed at this stage, patient enrollment was continued according to the original study plan to expand the sample size. **(B) Representative ultrasound scanning planes and corresponding images.** This figure presents four ultrasound scanning planes (left, demonstrated by the operator) alongside representative images (right). **(a)** Midsagittal view of the mandible: A low-frequency convex probe (Mindray TE7, C5-2s) was placed with the marker aligned with the chin and the opposite end over the superior border of the thyroid cartilage. **(b)** Transverse view of the hyoid bone: A high-frequency linear probe (Mindray TE7, L9-3s) was positioned laterally at a 45° angle to the horizontal plane. **(c)** Transverse view of the thyroid cartilage: The same probe was placed perpendicularly over the anterior neck to visualize the thyroid cartilage. **(d)** Paramedian sagittal view of the larynx: The ultrasound probe was placed 1 cm lateral to the neck midline, with its marked end 1 cm superior to the hyoid upper border, its plane parallel to the midline and angled at 45° to the horizontal plane. Key anatomical landmarks related to airway assessment, such as the hyoid bone and epiglottis, are shown in red. **(C) CL Classification. (D) VIDIAC scoring system used for video laryngoscopic classification.** (Created by the authors, based on concepts described in Kohse EK, et al. Anaesthesia. 2022. This image is similar to but not identical with the original one, and is therefore intended solely for illustrative purposes.) The VIDIAC system comprises three components: (1) Epiglottis – contact between the blade tip and epiglottis; (2) Vocal cords – best achievable view of the glottis; (3) Arytenoids – presence of hypertrophy. Scores range from –1 to 5. In this study, VIDIAC ≥3 was defined as a difficult airway. CL, Cormack–Lehane grade; VIDIAC, Videolaryngoscopic Intubation and Difficult Airway Classification.

This prospective study was approved by the Ethics Committee of the Affiliated Hospital of Qingdao University (Approval No.: QYFYEC2024−68) and registered at the Chinese Clinical Trial Registry (Registration No.: ChiCTR2400084274). All study participants provided written informed consent. The individual pictured in Fig 1 has provided written informed consent (as outlined in PLOS consent form) to publish their image alongside the manuscript. According to the research protocol, an interim analysis was conducted after 300 patients were enrolled to preliminarily assess model performance and determine whether the sample size was sufficient. As shown in the interim analysis results of this study (see S1 and S2 Tables), the model’s performance significantly decreased when applied to the validation set, with an AUC value below 0.75. This indicated that the model’s generalization ability was insufficient at this stage, suggesting that the initial sample size was inadequate for stable model training. Therefore, after obtaining approval from the ethics committee, patient recruitment continued, expanding the dataset by approximately three times to improve the model’s robustness and generalization ability. All other aspects remained consistent with the original research protocol.

### Data sets

Neck ultrasound images were collected from patients undergoing general anesthesia surgery at the Affiliated Hospital of Qingdao University from May 2024 to April 2025. The inclusion criteria were as follows: age ≥ 18 years; scheduled for elective surgery requiring tracheal intubation; willingness to participate in this study and provision of written informed consent. The exclusion criteria were as follows: presence of language communication disorders, neurological disorders, or inability to cooperate with ultrasound examination; scheduled for emergency surgery; complicated with head and neck fractures; not using neuromuscular blockers during intubation. Among these, 752 samples collected from May 2024 to February 2025 were used as the training and validation sets for constructing the difficult airway visualization model based on ultrasound images. The 151 samples collected from February 2025 to April 2025 were used as the internal test set to evaluate the classification performance of the predictive model. Detailed information on the number of excluded samples and data control is provided in Fig 1A.

### Ultrasound acquisition

Baseline characteristics, medical history, and results of conventional airway assessments were collected for all patients before surgery, including the Mallampati classification, head and neck mobility, upper lip bite test, mandibular horizontal length, thyromental distance, and interincisor gap. For each patient, we placed the individual in a supine position, with the neck extended to 45° (if 45°could not be achieved, the neck was extended to the maximum tolerable angle). Ultrasound examinations were performed at four predefined cervical scanning planes: (1) The midsagittal plane of the mandible was acquired using a low-frequency convex array transducer (C5–2s, Mindray TE7; 2–5 MHz); (2) The transverse plane of the hyoid bone, (3) The transverse plane of the thyroid cartilage, and (4) The paramedian sagittal plane of the larynx were obtained using a high-frequency linear array transducer (L9–3s, Mindray TE7; 3–9 MHz). Detailed instructions for ultrasound probe positioning and scanning standards can be found in the description of Fig 1B. Four ultrasound images were obtained per patient (one image per scanning plane). Depth settings: the midsagittal plane of the mandible is set to 8 cm, and the other three planes are all set to 4 cm. The gain was dynamically adjusted by the operator to ensure optimal image quality. All images were saved in PNG format. They were uniformly resized to a fixed input size (224 × 224 pixels) using proportional scaling and named according to the patient code. The names consist of the patient code and plane identifier (e.g., “Patient 1 Plane 2”), to distinguish between different patients and different plane images of the same patient. Conventional airway assessments were conducted by two resident physicians, while ultrasound assessments were independently performed by one ultrasonologist with over 10 years of clinical experience and two anesthesiologists with over 5 years of experience who had received specialized ultrasound training. All ultrasound examinations were carried out in strict accordance with standardized scanning protocols. None of these investigators were involved in subsequent anesthesia administration or difficult airway evaluation.

### Difficult airway labeling and reference standards

All patients underwent standardized vital sign monitoring prior to anesthesia induction. Anesthesia induction was performed using midazolam (2–3 mg), sufentanil (0.25–0.5 μg/kg), propofol (2–3 mg/kg), and rocuronium (0.8 mg/kg), followed by 3 minutes of mask ventilation. Tracheal intubation was conducted using a direct laryngoscope (Macintosh blade) and a video laryngoscope (Model: TD-C-IV; Manufacturer: Zhejiang Youyi Medical Device Co., Ltd.). The Cormack-Lehane (CL) classification [24] (Fig 1C) was used to assess difficult laryngoscopic exposure under DL, while The Videolaryngoscopic Intubation and Difficult Airway Classification (VIDIAC) scoring system [25] (Fig 1D) was used for assessment under VL. In this study, a score of ≥ 3 on either classification was defined as “difficult laryngoscopic exposure”. Two designated anesthesiologists were assigned to conduct airway assessments separately: one performed the CL classification assessment, while the other independently completed the VIDIAC assessment. Both were blinded to each other’s assessment results and to the results of the preoperative ultrasound and airway assessments. Both anesthesiologists were senior clinicians with more than 8 years of clinical experience and extensive experience in intubation with both DL and VL. If difficult tracheal intubation occurred, the chief anesthesiologist was consulted for management.

### Study workflow

**Fig 2:**
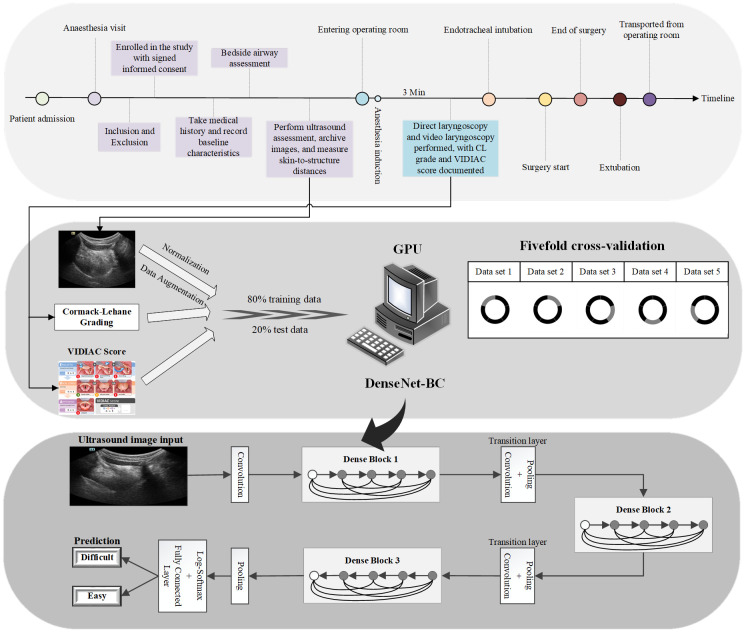
Schematic flowchart of study methodology. The diagram summarizes the overall study process: Top layer: perioperative data and image acquisition; Middle layer: training and validation of deep learning models; Bottom layer: DenseNet-BC architecture used for image-based prediction. GPU = Graphics Processing Unit.

This study used a transfer learning approach to evaluate the performance of 8 mainstream convolutional neural network (CNN) architectures in airway classification tasks based on ultrasound images. The Dense Convolutional Network-Bottleneck/Compression (DenseNet-BC) [26] performed the best (see S3 Table for details). This model alleviates gradient vanishing and improves gradient transfer efficiency and overall performance through its Bottleneck and Compression features. Finally, this study adopted a customized DenseNet (Fig 2) architecture. The network was composed of three dense blocks and their corresponding transition layers, incorporating bottleneck structures (a 1 × 1 convolution layer followed by a 3 × 3 convolution layer), batch normalization, and transition layer compression structures. The input of the model was single-channel grayscale ultrasound images. All ultrasound images were fed into the model in PNG format with a size of 224 × 224 pixels. Prior to model training, pixel intensity normalization was performed. Independent DenseNet sub-models were trained separately for each of the four ultrasound planes. We assigned weights to each sub-model based on the proportion of their average accuracy achieved in cross-validation. By integrating the prediction results of all planes, we developed two artificial intelligence models: the CL classification artificial intelligence model (CL-AI) and the VIDIAC score artificial intelligence model (VIDIAC-AI). The training configuration was as follows: cross-entropy loss function [27] was used; stochastic gradient descent (SGD) [28] optimizer (initial learning rate: 0.1, weight decay: 1e-4) was adopted; the learning rate decayed to 0.1 times its original value every 30 epochs; batch size: 64; total training epochs: 100. Given the severe class imbalance in the training set, an oversampling strategy was applied only within the training folds. Oversampling was achieved through image augmentation, including small-angle rotations (±15°) and brightness adjustments (±15%). No augmentation was applied to the internal test set. The model training was conducted using 5-fold cross-validation at the patient level: The patients included in the first 9 months were divided into training/validation sets in a 8:2 ratio, and there was no overlap of patients in each fold validation set to ensure that the model could be fully trained and to conduct a comprehensive evaluation of all patient samples. All ultrasound images of the same patient were assigned to the same subset. The patients included in the last 2 months were used as an independent internal test set and did not participate in the model training throughout the process.

### Statistical analysis

PyTorch 2.3.1 was used as the deep learning library, and Python 3.9.19 was adopted as the development environment. Additionally, the analytical hardware used included an Intel(R) Core(TM) i5-12600KF CPU, an NVIDIA GeForce RTX 4070 Super 12GB GPU, and Microsoft Windows 11 Professional Edition. All statistical analyses were performed using Python 3.9.19, and the results were presented as mean ± standard deviation (SD) and numbers (percentages). Receiver Operating Characteristic (ROC) curves were generated for the constructed models, and indicators including Area Under the Curve (AUC), accuracy, sensitivity, specificity, F1-score, and precision were calculated. The Bootstrap method was used to resample the internal test set for 1000 times, and the 95% confidence intervals of accuracy, sensitivity, specificity, F1-score and precision were calculated. The model was calibrated and analyzed, and the calibration curve was drawn to evaluate the consistency between the predicted probability of the model and the actual observation results.The Grad-CAM technique was used to visualize the model’s discriminative regions, so as to demonstrate the anatomical structures focused on by the models when distinguishing difficult airways. A model was considered to have sufficient diagnostic capability when its AUC > 0.70 and the lower bound of the 95% confidence interval (CI) > 0.50. To achieve the reproducibility of the research methodology, the implementation code and related descriptions of deep learning techniques in this study have all been made open-source and can be accessed online: https://doi.org/10.5281/zenodo.18140439.

## Results

### Dataset characteristics

A total of 1693 patients undergoing general anesthesia were screened between May 2024 and April 2025. Among them, 134 were excluded due to age < 18 years, 15 due to language communication disorders, 202 due to emergency surgery, 268 due to non-tracheal intubation anesthesia, 11 due to awake tracheal intubation, and 39 due to refusal to participate in the study. Subsequently, 1024 patients initially met the eligibility criteria. Additionally, 41 samples were excluded due to incomplete ultrasound plane scanning, 27 due to unfinished video laryngoscopy assessment, and 53 due to surgical cancellation. A total of 903 patients were finally included. Among them, 752 patients collected in the first 10 months (mean age: 59.4 ± 11.3 years; 293 females) were used for model training and validation, and 151 patients collected in the last 2 months (mean age: 57.9 ± 9.0 years; 65 females) were used for internal testing (see Table 1 for details). Detailed information on surgery-related details of this study is provided in S4 Table. Furthermore, under DL, 20.8% (157/752) of cases in the training cohort and 21.3% (32/151) of cases in the internal test cohort were classified as having difficult laryngoscopic exposure. Among patients undergoing VL, difficult laryngoscopic exposure cases were relatively rare, accounting for 5.6% (42/752) in the training cohort and 5.3% (8/151) in the internal test cohort. Due to sample size imbalance between patients with easy and difficult visualization, oversampling was performed on the training set data of each fold. For the DL training set (n = 600), there were 475 cases in the easy group and 125 cases in the difficult group; the sample size of the easy group was expanded to 1.5 times its original size, and that of the difficult group to 6 times its original size. For the VL training set (n = 600), there were 567 cases in the easy group and 33 cases in the difficult group; the sample size of the easy group was expanded to 1.5 times its original size, and that of the difficult group to 25 times its original size (see S5 Table for details).

**Table 1:** Patients’background characteristics.

Variable	Training Set	Internal Test Set
Total no. of patients	752	151
Height (cm)	167.2 ± 6.2	166.6 ± 5.0
Weight (Kg)	70.0 ± 9.7	69.4 ± 7.5
BMI (Kg/m^2^)	25.12 ± 2.7	25.10 ± 1.2
Age (years)	59.4 ± 11.3	57.9 ± 9.0
Sex (Male/Female)	293/459	65/86
ASA PS (1/2/3/4)	0/330/442/0	0/74/77/0
Hypertension (%)	287(38.2)	51(33.8)
Diabetes (%)	76(10.1)	12(7.95)
Mallampati Classification (MPC) (1/2/3/4)	317/223/154/58	60/47/33/11
Inter-incisor gap (IIG) (cm)	3.9 ± 0.5	3.86 ± 0.30
Head and neck movements (HNM) (>90°/ = 90°/ < 90°)	479/115/158	126/9/16
Thyromental distance (TMD) (cm)	7.5 ± 0.6	7.47 ± 0.23
Horizontal length of mandible (HLM) (cm)	12.7 ± 1.0	12.73 ± 0.54
ULBT (1/2/3)	435/214/103	86/43/22
Difficult laryngoscopic exposure under direct laryngoscopy (%)	157 (20.8)	32 (21.3)
Difficult laryngoscopic exposure under video laryngoscopy (%)	42 (5.6)	8(5.3)

Data are presented as mean ± standard deviation for continuous variables, number (percentage) for categorical variables, or number distribution for ordinal variables. Abbreviations: ASA PS, American Society of Anesthesiologists Physical Status Classification System; BMI, Body Mass Index; ULBT, Upper Lip Bite Test.

### Deep learning model performance

**Fig 3:**
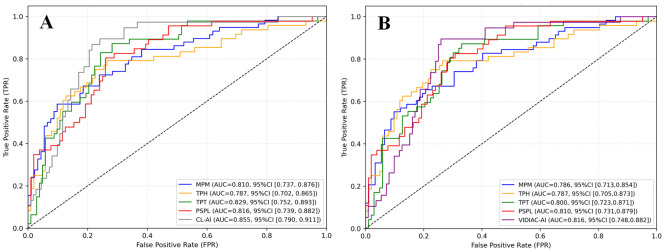
ROC curves of different AI models. **(A)** ROC curve comparison under the Cormack-Lehane (CL) classification framework, showing the performance of four ultrasound planes and the integrated model.(B) ROC curve comparison under the VIDIAC framework, showing the performance of four ultrasound planes and the integrated model. CL, Cormack–Lehane classification; VIDIAC, Videolaryngoscopic Intubation and Difficult Airway Classification; AI, artificial intelligence; DSH, Distance Skin to Hyoid; DSE, Distance Skin to Epiglottis; TMD, Thyromental Distance; others listed in figure.

After sample expansion, the training set was used for model training, the validation set for evaluating the internal robustness of the model, and the internal test set for final performance evaluation. The detailed results of the 5-fold cross-validation are shown in S6 Table. Finally, receiver operating characteristic (ROC) curves were generated based on the prediction results of each AI model on the test set (Fig 3), and metrics including area under the curve (AUC), accuracy, sensitivity, specificity, precision, and F1 score, along with their 95% confidence intervals [CI], were calculated. The results are shown in Table 2. In the CL classification task, the AUC values of the models for each ultrasound plane (MPM, TPH, TPT, PSPL) were 0.81 (0.74, 0.88), 0.79 (0.70, 0.87), 0.83 (0.75, 0.89), and 0.82 (0.74, 0.88), respectively, the accuracy ranged from 0.77 to 0.81, sensitivity from 0.75 to 0.81, specificity from 0.77 to 0.81, precision from 0.47 to 0.51, and F1 score from 0.58 to 0.65. The integrated CL-AI model showed better performance, with an AUC of 0.86 (0.79, 0.91), accuracy of 0.84, sensitivity of 0.84, specificity of 0.84, precision of 0.59, and F1 score of 0.69. In the VIDIAC assessment task, the AUC values of the models for each ultrasound plane (MPM, TPH, TPT, PSPL) were 0.79 (0.71, 0.85), 0.79 (0.70, 0.87), 0.80 (0.72, 0.87), and 0.81 (0.73, 0.89), with accuracy ranging from 0.77 to 0.79, sensitivity from 0.63 to 0.75, specificity from 0.77 to 0.80, precision from 0.13 to 0.16, and F1 score from 0.22 to 0.26. The AUC of the integrated VIDIAC-AI model was 0.82 (0.75, 0.88), the accuracy was 0.81, the sensitivity was 0.75, the specificity was 0.81, the precision was 0.18, and the F1 score was 0.29. All VIDIAC-based models exhibited relatively low precision and F1 scores, and all had wide confidence intervals for sensitivity. The calibration curves of the CL-AI and VIDIAC-AI models are presented in S1 Fig.

**Table 2:** Detailed parameters of the artificial intelligence prediction model in the internal test set.

Plane	AUC (95%CI)	Accuracy (95%CI)	Sensitivity (95%CI)	Specificity (95%CI)	Precision (95%CI)	F1 (95%CI)
**CL classification**						
MPM	0.81 (0.74, 0.88)	0.79 (0.71, 0.86)	0.75 (0.58, 0.89)	0.81 (0.72, 0.87)	0.51 (0.38, 0.63)	0.61 (0.50, 0.71)
TPH	0.79 (0.70, 0.87)	0.77 (0.70, 0.84)	0.75 (0.57, 0.88)	0.77 (0.70, 0.85)	0.47 (0.35, 0.60)	0.58 (0.47, 0.69)
TPT	0.83 (0.75, 0.89)	0.81 (0.74, 0.87)	0.81 (0.64, 0.92)	0.81 (0.73, 0.87)	0.53 (0.40, 0.65)	0.65 (0.54, 0.75)
PSPL	0.82 (0.74, 0.88)	0.79 (0.72, 0.86)	0.78 (0.61, 0.89)	0.80 (0.71, 0.86)	0.51 (0.38, 0.64)	0.62 (0.51, 0.73)
CL-AI	0.86 (0.79, 0.91)	0.84 (0.77, 0.90)	0.84 (0.68, 0.95)	0.84 (0.77, 0.91)	0.59 (0.46, 0.71)	0.69 (0.58, 0.78)
**VIDIAC**						
MPM	0.79 (0.71,0.85)	0.77 (0.71, 0.83)	0.75 (0.37, 0.96)	0.77 (0.66, 0.84)	0.15 (0.04, 0.30)	0.26 (0.10, 0.41)
TPH	0.79 (0.70, 0.87)	0.77 (0.70, 0.84)	0.63 (0.29, 0.90)	0.78 (0.70, 0.85)	0.13 (0.03, 0.28)	0.22 (0.08, 0.38)
TPT	0.80 (0.72, 0.87)	0.77 (0.72, 0.85)	0.75 (0.39, 0.97)	0.78 (0.74, 0.87)	0.16 (0.05, 0.32)	0.26 (0.11, 0.43)
PSPL	0.81 (0.73, 0.89)	0.79 (0.73, 0.87)	0.63 (0.22, 0.92)	0.80 (0.73, 0.86)	0.15 (0.04, 0.31)	0.24 (0.09, 0.40)
VIDIAC-AI	0.82 (0.75, 0.88)	0.81 (0.76, 0.89)	0.75 (0.38, 0.96)	0.81 (0.75, 0.89)	0.18 (0.06, 0.35)	0.29 (0.12, 0.45)

AUC: Area Under the Curve; 95%CI: 95% Confidence Interval; CL: Cormack–Lehane; VIDIAC: The Videolaryngoscopic Intubation and Difficult Airway Classification; MPM: the Midsagittal Plane of the Mandible; TPH: Transverse plane of the hyoid bone; TPT: Transverse plane of the thyroid cartilage; PSPL: Paramedian sagittal plane of the larynx; CL-AI refers to an integrated artificial intelligence model of four models under direct laryngoscopy; VIDIAC-AI refers to an integrated artificial intelligence model of four models under video laryngoscopy.

### Comparison with anesthesiologists’ assessment performance

To assess the clinical applicability of the proposed models, the performance of AI models and anesthesiologists was compared on an internal test set (n = 151), where three junior (1–3 years of experience) and three senior (10–15 years) anesthesiologists independently conducted preoperative difficult airway assessments using routine clinical data (Includes physical examination, past medical history, and routine airway assessment measures, but no ultrasound examination), with predictions aggregated via majority voting for each group. In the DL scenario, juniors achieved an accuracy of 0.808, sensitivity of 0.781 and specificity of 0.815, seniors showed superior performance with an accuracy of 0.868, sensitivity of 0.875 and specificity of 0.866, and the CL-AI model reached an accuracy of 0.841, sensitivity of 0.844 and specificity of 0.840, which was on par with seniors, with paired McNemar’s tests showing no statistically significant differences between CL-AI and either group (all P > 0.05). In the VL scenario, seniors again delivered the best overall performance with an accuracy of 0.821, sensitivity of 0.750 and specificity of 0.825, juniors had moderate accuracy (0.782) but low sensitivity (0.500), the VIDIAC-AI model attained an accuracy of 0.808, sensitivity of 0.750 and specificity of 0.811, and McNemar’s tests indicated no statistically significant differences between VIDIAC-AI and either group (all P > 0.05) (see Table 3 for details).

**Table 3:** Performance comparison between anesthesiologists and AI models using direct and video laryngoscopy.

laryngoscope	Object	Accuracy	Sensitivity	Specificity	Precision	F1
**Direct laryngoscope**	Junior Anesthesiologist^*a^	0.808	0.781	0.815	0.531	0.633
	Senior Anesthesiologist^*b^	0.868	0.875	0.866	0.636	0.738
	CL-AI	0.841	0.844	0.840	0.587	0.690
**Video laryngoscope**	Junior Anesthesiologist^#a^	0.782	0.50	0.797	0.121	0.195
	Senior Anesthesiologist^#b^	0.821	0.75	0.825	0.194	0.307
	VIDIAC-AI	0.808	0.75	0.811	0.182	0.293

Performance comparison of junior anesthesiologists, senior anesthesiologists, and AI models in predicting difficult airway using direct laryngoscopy (CL) and video laryngoscopy (VIDIAC). Performance metrics include accuracy, sensitivity, specificity, F1 score, and precision. Junior anesthesiologist refers to an anesthesiologist with 3 years of clinical experience, whereas senior anesthesiologist refers to an anesthesiologist with 10 or more years of clinical experience. For comparisons involving anesthesiologists, predictions from three anesthesiologists within the same experience group were aggregated into a single group-level decision using a majority-vote strategy on a per-case basis. All evaluations were performed on the same independent test set. Paired comparisons between anesthesiologist groups and AI models were conducted at the patient level using McNemar’s test.

*a: McNemar test comparing junior anesthesiologist and CL-AI (χ² = 0, P = 1).

*b: McNemar test comparing senior anesthesiologist and CL-AI (χ² = 0.455, P = 0.831).

#a: McNemar test comparing junior anesthesiologist and VIDIAC-AI (χ² = 0.1, P = 0.752).

#b: McNemar test comparing senior anesthesiologist and VIDIAC-AI (χ² = 0.029, P = 0.864).

### Construction and visual explanation of the “two-model, three-step” decision-making system

**Fig 4:**
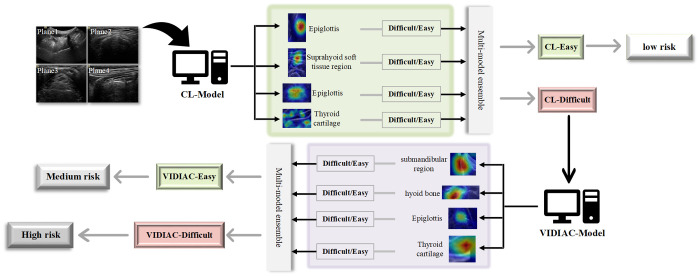
Schematic diagram of the artificial intelligence model workflow for difficult laryngoscopy prediction. The workflow illustrates the process of two AI models (CL-Model and VIDIAC-Model) analyzing four ultrasound planes to predict difficult laryngoscopy and stratify risk. Ultrasound images from four planes are input into the CL-Model, which generates attention heatmaps for regions like the epiglottis, suprahyoid soft tissue, and thyroid cartilage. A multi-model ensemble integrates these outputs to classify cases as CL-Easy (low risk) or CL-Difficult. For cases classified as CL-grade difficult airway, the VIDIAC model was further employed to analyze the ultrasound planes, focusing on regions such as the submandibular region, hyoid bone, epiglottis, and thyroid cartilage. Its multi-model ensemble classifies cases into VIDIAC-Easy (medium risk) or VIDIAC-Difficult (high risk), completing the risk stratification. CL, Cormack–Lehane classification; VIDIAC, Videolaryngoscopic Intubation and Difficult Airway Classification; AI, artificial intelligence.

Fig 4 illustrates the decision-making process using a visual heatmap and further demonstrates the advantages of the AI models. By integrating the CL-AI model and the VIDIAC-AI model, the “two-model, three-step” decision-making system was constructed. First, the four ultrasound plane images are input into the CL-AI model for analysis; the model identifies anatomical structures such as the epiglottis, suprahyoid soft tissue region, and thyroid cartilage; finally, through multi-model ensemble weighting, it outputs “CL-Easy” (low risk) or “CL-Difficult”. If the output is “CL-Difficult”, the ultrasound images are input into the VIDIAC-AI model. This model analyzes anatomical regions such as the submandibular region, hyoid bone, epiglottis, and thyroid cartilage, and finally outputs “VIDIAC-Easy” (moderate risk) or “VIDIAC-Difficult” (high risk) through weighting.

## Discussion

This study combines ultrasound imaging with deep learning techniques to predict difficult laryngoscopic exposure under DL and VL, and further proposes a two-model, three-step decision framework for perioperative airway risk stratification. In this framework, the CL-AI model showed good discrimination performance in identifying difficult DL exposure, with an AUC of 0.86 (95%CI: 0.79–0.91), accuracy of 0.84, sensitivity of 0.84, specificity of 0.84, precision of 0.59, and F1 score of 0.69. The VIDIAC-AI model had a good performance in discriminating difficult VL exposures (AUC: 0.82, 95%CI: 0.75–0.88), with an accuracy of 0.81, a sensitivity of 0.75, and a specificity of 0.81, but its precision (0.18) and F1 score (0.29) were relatively low. These findings suggest that the applicable scenario of the VIDIAC-AI model should be limited to subsequent risk restratification of the CL-AI model positive population, rather than considering it as an independent diagnostic tool.

The greatest challenge of this study was the relatively low incidence of difficult video laryngoscope exposure, which was 5.6% (42/752) in the training cohort and 5.3% (8/151) in the internal testing cohort. Therefore, in order to ensure that the model can fully learn the features of difficult cases in the training process, this study adopted a more radical oversampling strategy, which oversampled the difficult images in the training set under VL by 25 times. We also clearly recognize that this strategy may increase the risk of model overfitting if the number of positive samples is limited. Several control measures were implemented to mitigate this risk. First, oversampling and data augmentation were only implemented in the training fold of the five-fold cross validation, and the internal test set was not augmented in any way. Second, all data partitioning and enhancement were completed at the patient level to avoid the relevant images of the same patient appearing in different data sets, thereby preventing data leakage. Thirdly, multi-dimensional metrics including AUC, calibration curve and confidence interval were used to evaluate the performance of the model, instead of relying on accuracy alone. In addition, the oversampling method used in this study was not simply to repeat the sample, but to randomly rotate the sample within a range of ±15° and adjust the brightness within a range of ±15% to simulate the subtle differences in the Angle of the probe between different operators and the differences in ultrasound gain Settings in different examinations during ultrasound acquisition. No cropping-based or synthetic interpolation methods (e.g., mixup or CutMix) were applied to avoid distortion of anatomical structures. Nonetheless, the possibility of residual overfitting cannot be completely ruled out, so the results of the VL classification in this study should be regarded as a proof of concept. In the future, external validation in larger and multi-ethnic populations is needed to further confirm the robustness and clinical applicability of the framework. In addition, this severe class imbalance problem limits the statistical power of model training and evaluation. This resulted in a wide confidence interval for the sensitivity estimates of the VIDIAC-AI model. Although its AUC value was acceptable, the model still exhibited low precision and F1-score, with its calibration curve deviating from the ideal diagonal line. These results indicate that while the VIDIAC-AI model exhibits reasonable discriminative power, the reliability of positive predictions is still limited due to the small number of difficult video laryngoscopy events. The generalization power of the VIDIAC-AI model in this study should not be overestimated, so the model is not used as a stand-alone diagnostic tool in this study, but only as a secondary risk stratification in a multi-stage workflow, applied only to patients who have been previously identified as difficult by the CL-AI model.

Although the overall model performance of different ultrasound planes was similar, slight differences were observed. In DL and VL, the transverse plane above the thyroid cartilage (epiglottis plane) had the best performance, AUC under DL was 0.83, accuracy was 0.81, sensitivity was 0.81, specificity was 0.81. AUC under VL was 0.80, accuracy was 0.77, sensitivity was 0.75, specificity was 0.78. Ultrasound can provide abundant anatomical information related to the patient’s cervical airway, including key structures such as the hyoid bone, epiglottis, and mandible [12]. Ultrasound has been widely used in difficult airway assessment in the past [13,29,30]; for example, the distance from the skin to the epiglottis [31,32] and from the skin to the hyoid bone [15,33] has been measured to predict difficult airways. Among the various parameters, the distance from the skin to the epiglottis showed the most prominent predictive performance (AUC 0.87, sensitivity 78%, specificity 87% [13]), which is consistent with our research findings. Furthermore, the model visualization heatmap focused on these anatomical structures (epiglottis, hyoid bone, etc.), indicating a correlation between these structures and difficult laryngoscopy exposure. Our weighted CL-AI and VIDIAC-AI models, incorporating four different planes, performed better than single-plane models, suggesting that difficult laryngoscopy exposure is not only related to a single anatomical structure but is also a comprehensive result of the synergistic interaction of multiple anatomical structures. By combining ultrasound imaging with deep learning, our method aims to utilize information related to anatomical structures while reducing subjectivity and improving reproducibility. Importantly, our proposed framework is applicable to both DL and VL and supports a two-stage risk stratification strategy, which surpasses the binary prediction commonly found in previous studies.

In recent years, deep learning-based methods have been increasingly used for preoperative airway assessment. Several studies have reported promising results in predicting difficult laryngoscopy using facial image analysis [18–21] and laryngeal voice signals [22,23], with AUC ranging from 0.709 to 0.918, sensitivity from 0.737 to 0.875, and specificity from 0.63 to 0.833. However, these methods primarily rely on indirect or surrogate indicators of airway anatomy and cannot directly observe the underlying airway structures. Furthermore, most existing AI-based prediction models focus only on DL, limiting their application in VL scenarios and preventing risk stratification across different laryngoscopy settings. Multi-view and multi-modal fusion has emerged as an important direction in difficult airway prediction, demonstrating significantly improved quantitative performance compared to traditional methods. For example, Mannan Abdul et al [34] proposed a three-stage intelligent framework that integrates high-resolution facial images, infrared depth maps, and patient baseline clinical data, utilizing an XGBoost model, achieving a final AUC of 0.831. RMP-Net [35] fused keypoint maps and laryngoscopic prototype features, achieving an AUC of 85.07% and improving specificity to 83.18%, demonstrating the advantages of multi-modal features in enhancing discriminative ability. However, these methods typically rely on specialized hardware, complex acquisition procedures, and patient cooperation, and require additional training, limiting their clinical applicability. In contrast, this study employs a single-modal AI framework based on ultrasound, which can directly obtain deep anatomical information related to the airway while avoiding facial data collection, reducing privacy risks and deployment barriers. This provides a feasible and practical solution for current clinical practice and can serve as a complement to or fundamental component of future multi-modal systems.

**Fig 5:**
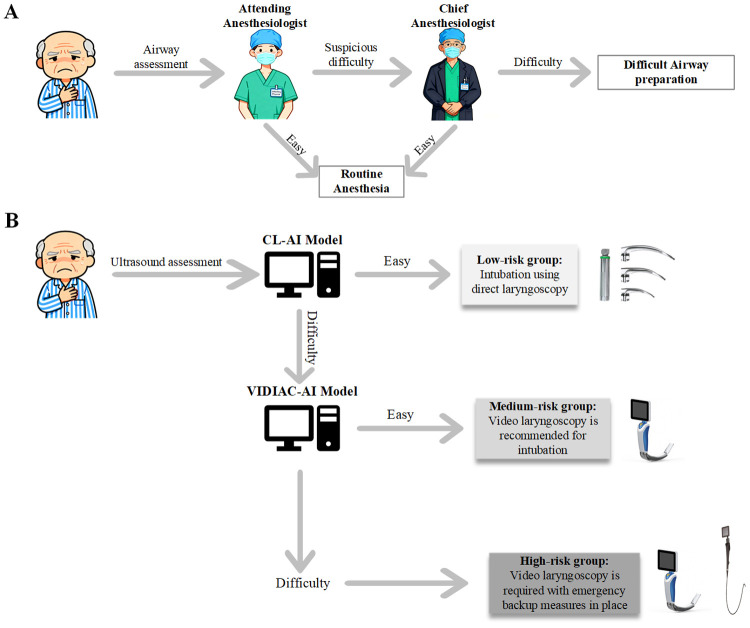
Comparison of airway assessment workflows. **(A)** Traditional workflow: Patient undergoes airway assessment by Attending and Chief Anesthesiologists, with difficult cases undergoing special preparation and easy cases receiving routine anesthesia. **(B)** AI-assisted workflow: Patient undergoes ultrasound assessment via CL-AI and VIDIAC-AI models, stratified into low-risk (direct laryngoscopy), medium-risk (video laryngoscopy), and high-risk (video laryngoscopy + emergency backup) groups. CL, Cormack–Lehane; VIDIAC, Videolaryngoscopic Intubation and Difficult Airway Classification; AI, artificial intelligence.

Furthermore, previous studies have mostly focused on difficult laryngoscopic exposure under DL [18,22,23,36,37], while studies on difficult laryngoscopic exposure under VL are relatively scarce. Additionally, previous VL-related studies have continued to use the traditional CL classification as the airway assessment standard. The operating principle of VL is fundamentally different from that of DL: VL can directly display the field of view at the root of the laryngoscope, effectively addressing the issue of insufficient visualization of the oropharyngeal-laryngeal field in DL [6,38]. Consequently, VL typically provides superior glottic visualization. The difficult exposure rate under VL reported in previous studies ranges from 2% to 5% [6,39,40], which is consistent with the results of this study. Given that glottis visualization is more distinct under VL, and that the degree of laryngoscopic exposure is also dependent on the relative positional relationship between the laryngoscope blade and the epiglottis. Therefore, the VIDIAC score was adopted in this study as the criterion for evaluating difficult laryngoscopic exposure under VL. As shown in Fig 5, by constructing models for difficult laryngoscopic exposure under DL and VL, we established the “two-model, three-step” decision-making system. The traditional process (Fig 5A) relies on the experience of anesthesiologists: airway conditions are judged through multi-level review by attending and chief anesthesiologists. This process is susceptible to variations in experience and may lead to delayed decision-making, especially when junior attending physicians are on duty independently. In contrast, the “two-model, three-step” decision-making system (Fig 5B) conducts hierarchical assessment using ultrasound combined with AI models: the CL-AI model classifies “Easy” as low risk, indicating intubation with DL; if it classifies “Difficult”, a re-evaluation is performed using the VIDIAC-AI model. “Easy” in this re-evaluation is classified as moderate risk, suggesting intubation with VL; “Difficult” is classified as high risk, requiring VL and preparation of advanced airway devices. Furthermore, in terms of various evaluation metrics, the model constructed in this study performed better than junior anesthesiologists and only slightly worse than senior anesthesiologists. However, it should be noted that the McNemar test results comparing anesthesiologists and the AI model showed that the P-values for all groups were greater than 0.05, meaning that the performance differences between the two could not be ruled out as being due to chance. The potential advantage of the “two-model, three-step” decision system constructed in this study lies in its preliminary approach to classifying difficult airways based on direct/video laryngoscopy and stratifying patient airway risk according to different prediction results. However, due to the limited sample size and single-center nature of this study, the applicability and stability of this decision system require further exploration and validation in larger, multi-center studies.

This study has the following strengths: First, it adopted the VIDIAC score, which is suitable for difficult airway assessment under VL, as the evaluation criterion instead of continuing to use the traditional CL classification. This effectively fills the research gap in the application of assessment criteria for difficult airways under VL. Second, this study systematically explored the application value of combining ultrasound technology with deep learning in predicting difficult laryngoscopic exposure, constructed the “two-model, three-step” decision-making system, and proposed the concept of precision airway management; this system is expected to facilitate the clinical implementation of personalized airway management in the future, thereby reducing the incidence of airway-related complications.

This study also has the following limitations: First, the study population only included patients undergoing elective surgery, and the assessment of difficult laryngoscopic exposure was performed after general anesthesia. In contrast, patients requiring awake intubation in the emergency department or ICU have different airway visualization conditions from the population in this study and may face greater difficulty in visualization. Second, for patients predicted to have a difficult airway through preoperative assessment, awake intubation is often selected in clinical practice; these patients did not undergo the post-anesthesia visualization assessment specified in this study, resulting in the exclusion of some potential cases of difficult visualization from the study. Third, this study was a single-center study; model validation only used an internal test set, and no external validation was conducted, which results in regional limitations in the constructed AI models. Furthermore, the incidence of difficult visualization under VL is inherently low, resulting in a relatively small sample size of difficult visualization cases in this study. This led to the VIDIAC-AI model exhibiting relatively low accuracy and wide confidence intervals. Future studies should adopt a multi-center, large-sample design to further validate the model’s generalization ability. Additionally, the scope of difficult airways includes difficult ventilation, difficult laryngoscopic exposure, and difficult tracheal intubation; this study only focused on the single dimension of difficult laryngoscopic exposure, leading to certain limitations in the research scope, and thus the conclusions cannot cover the complete assessment needs for difficult airways. Finally, given that airway management is a high-risk clinical decision, the AI framework presented in this study is intended as a clinical decision support tool, not a replacement for the professional judgment of anesthesiologists. The ultimate decision regarding airway management should remain the responsibility of the clinician, based on a comprehensive assessment combining AI results, clinical context, and real-time evaluation. It is particularly important to note that model misjudgments (especially false negative predictions) can have serious consequences. Therefore, manual supervision and careful interpretation of the model output are essential.

## Conclusion

Based on the CNN deep learning algorithm, this study developed an AI classification model that integrates features derived from four ultrasound planes for predicting difficult laryngoscopic exposure, and further constructed a Two-Model, Three-Step airway management decision framework. The results suggest that combining ultrasound images with deep learning methods may provide valuable information for preoperative airway risk assessment. At present, this system serves solely as a decision-support tool to assist clinicians, rather than an independent diagnostic method. Prior to its widespread clinical application, external validation and further model performance optimization remain necessary in larger-scale, multicenter cohorts.

## Supporting information

S1 Table Taking the Transverse plane of the hyoid bone under direct laryngoscopy as an example, the performance comparison between the validation set and the internal test set. The AUC (95% Confidence Interval, 95% CI) of the deep learning model was set as the primary performance metric, with accuracy, sensitivity, and specificity as the secondary metrics. The AUC of all AI models on the test set was < 0.75, indicating insufficient generalizability of the models at present. When the model AUC is < 0.75, the sample size needs to be expanded to at least 3–4 times the initial size to improve the model’s generalizability. Under the framework of the original study plan, this study continuously enrolled eligible patients, and a total of 903 cases were finally collected. All patients signed the informed consent form.
(DOCX)

S2 Table Patient Baseline Characteristics of the Interim Analysis Cohort and the Final Cohort. Data are presented as mean ± standard deviation for continuous variables, number (percentage) for categorical variables, or number distribution for ordinal variables. Abbreviations: ASA PS, American Society of Anesthesiologists Physical Status Classification System; BMI, Body Mass Index; ULBT, Upper Lip Bite Test.
(DOCX)

S3 Table The performance of eight mainstream convolutional neural networks (CNNS) in the pre-experimental test set. During the interim analysis, we used 5-fold cross-validation (Dataset1–Dataset5 represent the 5 folds) to evaluate the performance of eight mainstream convolutional neural networks (CNNs) in the pre-experimental test set. The model with the best performance, identified as DenseNet-BC*, is marked with an asterisk.
(DOCX)

S4 Table Distribution of surgical procedures in patients. (DOCX)

S5 Table Sample size data enhancement details of the datasets of Direct laryngoscope and Video laryngoscope. In the cross-validation of AI models under direct laryngoscopy and video laryngoscopy, 80% of the data was used as the training set, and 20% was designated as the validation set. The table details the sample size distribution across the 5 cross-validation folds (dataset1–dataset5) for both direct laryngoscopy and video laryngoscopy, including the training data before and after data expansion.
(DOCX)

S6 Table Mean performance of individual ultrasound planes for CL and VIDIAC classification obtained from five-fold cross-validation. Values are reported as mean ± standard deviation across the five folds. AUC and accuracy were calculated independently within each fold and then averaged. Abbreviations: AUC: Area Under the Curve; SD: Standard Deviation; CL: Cormack–Lehane; VIDIAC: The Videolaryngoscopic Intubation and Difficult Airway Classification; MPM: the Midsagittal Plane of the Mandible; TPH: Transverse plane of the hyoid bone; TPT: Transverse plane of the thyroid cartilage; PSPL: Paramedian sagittal plane of the larynx; CL-AI refers to an integrated artificial intelligence model of four models under direct laryngoscopy; VIDIAC-AI refers to an integrated artificial intelligence model of four models under video laryngoscopy.
(DOCX)

S1 Fig Calibration plots of the AI models in the independent test set. (A) Calibration plot of the CL-AI model. (B) Calibration plot of the VIDIAC-AI model. Predicted probabilities were grouped into five bins according to the mean predicted risk. The solid line with points represents the mean observed proportion of difficult airway events within each bin, and the shaded area indicates the 95% confidence interval estimated using 1,000 bootstrap resamples. The dashed diagonal line represents perfect calibration (y = x). Wider uncertainty and deviations from the ideal line, particularly for the VIDIAC-AI model, are expected given the limited number of positive cases in the test set.
(TIF)
