# Supplementary material for: An ultrasound-based artificial intelligence framework for difficult airway prediction: A two-model, three-step decision framework
Source: PLoS One. 2026 Feb 18;21(2):e0342339. doi: 10.1371/journal.pone.0342339 (PMC12915933; doi:10.1371/journal.pone.0342339)
Supplement: S1 Table — The AUC (95% Confidence Interval, 95% CI) of the deep learning model was set as the primary performance metric, with accuracy, sensitivity, and specificity as the secondary metrics. The AUC of all AI models on the test set was < 0.75, indicating insufficient generalizability of the models at present. When the model AUC is < 0.75, the sample size needs to be expanded to at least 3–4 times the initial size to improve the model’s generalizability. Under the framework of the original study plan, this study continuously enrolled eligible patients, and a total of 903 cases were finally collected. All patients signed the informed consent form. (DOCX) [file pone.0342339.s001.docx]

From May to August 2024, this study collected 300 cases, followed by an interim analysis. The purpose was to evaluate the preliminary performance of the model using phased data and ensure that the sample size meets the research requirements.

**S1 Table. Taking the Transverse plane of the hyoid bone under direct laryngoscopy as an example, the performance comparison between the validation set and the internal test set.**

| **Performance Metrics** | **Validation Set（N=50）** | **Test Set（N=50）** |
| --- | --- | --- |
| AUC（95%CI） | 0.89 (0.86, 0.92) | 0.65 (0.57, 0.68) |
| Accuracy (%) | 0.81 | 63.3 |
| Sensitivity (%) | 0.81 | 63.6 |
| Specificity (%) | 0.82 | 63.1 |

The AUC (95% Confidence Interval, 95% CI) of the deep learning model was set as the primary performance metric, with accuracy, sensitivity, and specificity as the secondary metrics. The AUC of all AI models on the test set was < 0.75, indicating insufficient generalizability of the models at present. When the model AUC is < 0.75, the sample size needs to be expanded to at least 3–4 times the initial size to improve the model's generalizability. Under the framework of the original study plan, this study continuously enrolled eligible patients, and a total of 903 cases were finally collected. All patients signed the informed consent form.
