# Supplementary material for: An ultrasound-based artificial intelligence framework for difficult airway prediction: A two-model, three-step decision framework
Source: PLoS One. 2026 Feb 18;21(2):e0342339. doi: 10.1371/journal.pone.0342339 (PMC12915933; doi:10.1371/journal.pone.0342339)
Supplement: S2 Table — Data are presented as mean ± standard deviation for continuous variables, number (percentage) for categorical variables, or number distribution for ordinal variables. Abbreviations: ASA PS, American Society of Anesthesiologists Physical Status Classification System; BMI, Body Mass Index; ULBT, Upper Lip Bite Test. (DOCX) [file pone.0342339.s002.docx]

**S2 Table. Patient Baseline Characteristics of the Interim Analysis Cohort and the Final Cohort.**

| **Variable** | **Interim Analysis Cohort** | **Final Cohort** | |
| --- | --- | --- | --- |
|  |  | **Training Set** | **Internal Test Set** |
| Total no. of patients | 300 | 752 | 151 |
| Height (cm) | 167.3 ± 6.5 | 167.2 ± 6.2 | 166.6 ± 5.0 |
| Weight (Kg) | 70.4 ± 10.5 | 70.0 ± 9.7 | 69.4 ± 7.5 |
| BMI (Kg/m2) | 25.1 ± 3.1 | 25.12 ± 2.7 | 25.10 ± 1.2 |
| Age (years) | 58.9 ± 12.2 | 59.4 ± 11.3 | 57.9 ± 9.0 |
| Sex (Male/Female) | 115/185 | 293/459 | 65/86 |
| ASA PS (1/2/3/4) | 0/127/173/0 | 0/330/442/0 | 0/74/77/0 |
| Hypertension (%) | 189(63) | 287(38.2) | 51(33.8) |
| Diabetes (%) | 266(88.7) | 76(10.1) | 12(7.95) |
| Mallampati Classification (MPC) (1/2/3/4) | 162/100/28/10 | 317/223/154/58 | 60/47/33/11 |
| Inter-incisor gap (IIG) (cm) | 3.9 ± 0.6 | 3.9 ± 0.5 | 3.86 ± 0.30 |
| Head and neck movements (HNM) (>90°/=90°/<90°) | 215/65/15 | 479/115/158 | 126/9/16 |
| Thyromental distance (TMD) (cm) | 7.5 ± 0.7 | 7.5 ± 0.6 | 7.47 ± 0.23 |
| Horizontal length of mandible (HLM) (cm) | 12.7 ± 1.1 | 12.7 ± 1.0 | 12.73 ± 0.54 |
| ULBT (1/2/3) | 243/51/6 | 435/214/103 | 86/43/22 |
| Difficult laryngoscopic exposure under direct laryngoscopy (%) | 66 (22) | 157 (20.8) | 32 (21.3) |
| Difficult laryngoscopic exposure under video laryngoscopy (%) | 19 (6.3) | 42 (5.6) | 8(5.3) |

Data are presented as mean ± standard deviation for continuous variables, number (percentage) for categorical variables, or number distribution for ordinal variables. Abbreviations: ASA PS, American Society of Anesthesiologists Physical Status Classification System; BMI, Body Mass Index; ULBT, Upper Lip Bite Test.
