# Supplementary material for: An ultrasound-based artificial intelligence framework for difficult airway prediction: A two-model, three-step decision framework
Source: PLoS One. 2026 Feb 18;21(2):e0342339. doi: 10.1371/journal.pone.0342339 (PMC12915933; doi:10.1371/journal.pone.0342339)
Supplement: S3 Table — During the interim analysis, we used 5-fold cross-validation (Dataset1–Dataset5 represent the 5 folds) to evaluate the performance of eight mainstream convolutional neural networks (CNNs) in the pre-experimental test set. The model with the best performance, identified as DenseNet-BC*, is marked with an asterisk. (DOCX) [file pone.0342339.s003.docx]

**S3 Table. The performance of eight mainstream convolutional neural networks (CNNS) in the pre-experimental test set.**

| **Model** | **Dataset1** | **Dataset2** | **Dataset3** | **Dataset4** | **Dataset5** | **Average** |
| --- | --- | --- | --- | --- | --- | --- |
| Vgg16 | 0.758 | 0.903 | 0.576 | 0.947 | 0.944 | 0.8256 |
| ResNet | 0.958 | 0.903 | 0.782 | 0.959 | 0.928 | 0.8804 |
| AlexNet | 0.705 | 0.857 | 0.688 | 0.788 | 0.928 | 0.7932 |
| MobileNet | 0.905 | 0.878 | 0.847 | 0.858 | 0.939 | 0.8854 |
| EfficientNet | 0.921 | 0.697 | 0.571 | 0.391 | 0.828 | 0.6816 |
| EfficientNetV2 | 0.895 | 0.504 | 0.465 | 0.476 | 0.683 | 0.6046 |
| DenseNet | 0.88 | 0.85 | 0.76 | 0.86 | 0.76 | 0.822 |
| **DenseNet-BC*** | **0.93** | **0.9** | **0.95** | **0.85** | **0.81** | **0.888** |

During the interim analysis, we used 5-fold cross-validation (Dataset1–Dataset5 represent the 5 folds) to evaluate the performance of eight mainstream convolutional neural networks (CNNs) in the pre-experimental test set. The model with the best performance, identified as DenseNet-BC*, is marked with an asterisk.
