# Supplementary material for: An ultrasound-based artificial intelligence framework for difficult airway prediction: A two-model, three-step decision framework
Source: PLoS One. 2026 Feb 18;21(2):e0342339. doi: 10.1371/journal.pone.0342339 (PMC12915933; doi:10.1371/journal.pone.0342339)
Supplement: S4 Table — (DOCX) [file pone.0342339.s004.docx]

**S4 Table. Distribution of surgical procedures in patients.**

| **Surgery Department** | **Number of Patients** |
| --- | --- |
| Oral and maxillofacial surgery | 227 |
| Urology | 216 |
| Vascular surgery | 179 |
| Gastrointestinal surgery | 136 |
| Gynecology | 145 |
