# Supplementary material for: An ultrasound-based artificial intelligence framework for difficult airway prediction: A two-model, three-step decision framework
Source: PLoS One. 2026 Feb 18;21(2):e0342339. doi: 10.1371/journal.pone.0342339 (PMC12915933; doi:10.1371/journal.pone.0342339)
Supplement: S5 Table — In the cross-validation of AI models under direct laryngoscopy and video laryngoscopy, 80% of the data was used as the training set, and 20% was designated as the validation set. The table details the sample size distribution across the 5 cross-validation folds (dataset1–dataset5) for both direct laryngoscopy and video laryngoscopy, including the training data before and after data expansion. (DOCX) [file pone.0342339.s005.docx]

**S5 Table. Sample size data enhancement details of the datasets of Direct laryngoscope and Video laryngoscope.**

|  | **Validation set** | | **Training data** | | **Training data after data expansion** | |
| --- | --- | --- | --- | --- | --- | --- |
|  | Easy | Difficult | Easy | Difficult | Easy | Difficult |
| Direct laryngoscope | | | | | | |
| dataset1 | 119 | 32 | 475 | 125 | 720 | 750 |
| dataset2 | 119 | 32 | 475 | 125 | 720 | 750 |
| dataset3 | 119 | 32 | 475 | 125 | 720 | 750 |
| dataset4 | 119 | 32 | 475 | 125 | 720 | 750 |
| dataset5 | 119 | 32 | 475 | 125 | 720 | 750 |
| Video laryngoscope | | | | | | |
| dataset1 | 134 | 17 | 567 | 33 | 850 | 825 |
| dataset2 | 134 | 17 | 567 | 33 | 850 | 825 |
| dataset3 | 134 | 17 | 567 | 33 | 850 | 825 |
| dataset4 | 134 | 17 | 567 | 33 | 850 | 825 |
| dataset5 | 134 | 17 | 567 | 33 | 850 | 825 |

In the cross-validation of AI models under direct laryngoscopy and video laryngoscopy, 80% of the data was used as the training set, and 20% was designated as the validation set. The table details the sample size distribution across the 5 cross-validation folds (dataset1–dataset5) for both direct laryngoscopy and video laryngoscopy, including the training data before and after data expansion.
