# Supplementary material for: An ultrasound-based artificial intelligence framework for difficult airway prediction: A two-model, three-step decision framework
Source: PLoS One. 2026 Feb 18;21(2):e0342339. doi: 10.1371/journal.pone.0342339 (PMC12915933; doi:10.1371/journal.pone.0342339)
Supplement: S6 Table — Values are reported as mean ± standard deviation across the five folds. AUC and accuracy were calculated independently within each fold and then averaged. Abbreviations: AUC: Area Under the Curve; SD: Standard Deviation; CL: Cormack–Lehane; VIDIAC: The Videolaryngoscopic Intubation and Difficult Airway Classification; MPM: the Midsagittal Plane of the Mandible; TPH: Transverse plane of the hyoid bone; TPT: Transverse plane of the thyroid cartilage; PSPL: Paramedian sagittal plane of the larynx; CL-AI refers to an integrated artificial intelligence model of four models under direct laryngoscopy; VIDIAC-AI refers to an integrated artificial intelligence model of four models under video laryngoscopy. (DOCX) [file pone.0342339.s006.docx]

**S6 Table. Mean performance of individual ultrasound planes for CL and VIDIAC classification obtained from five-fold cross-validation.**

| **Plane** | **Mean AUC ± SD** | **Mean Accuracy ± SD** |
| --- | --- | --- |
| CL classification | | |
| MPM | 0.89±0.01 | 0.84±0.02 |
| TPH | 0.88±0.02 | 0.85±0.03 |
| TPT | 0.90±0.02 | 0.86±0.02 |
| PSPL | 0.89±0.03 | 0.85±0.03 |
| VIDIAC | | |
| MPM | 0.87±0.03 | 0.83±0.03 |
| TPH | 0.89±0.02 | 0.84±0.03 |
| TPT | 0.88±0.02 | 0.85±0.02 |
| PSPL | 0.87±0.03 | 0.84±0.04 |

Values are reported as mean ± standard deviation across the five folds. AUC and accuracy were calculated independently within each fold and then averaged.

AUC: Area Under the Curve; SD: Standard Deviation; CL: Cormack–Lehane; VIDIAC: The Videolaryngoscopic Intubation and Difficult Airway Classification; MPM: the Midsagittal Plane of the Mandible; TPH: Transverse plane of the hyoid bone; TPT: Transverse plane of the thyroid cartilage; PSPL: Paramedian sagittal plane of the larynx; CL-AI refers to an integrated artificial intelligence model of four models under direct laryngoscopy; VIDIAC-AI refers to an integrated artificial intelligence model of four models under video laryngoscopy.
