# Supplementary figures and images for: An ultrasound-based artificial intelligence framework for difficult airway prediction: A two-model, three-step decision framework
Source: PLoS One. 2026 Feb 18;21(2):e0342339. doi: 10.1371/journal.pone.0342339 (PMC12915933; doi:10.1371/journal.pone.0342339)

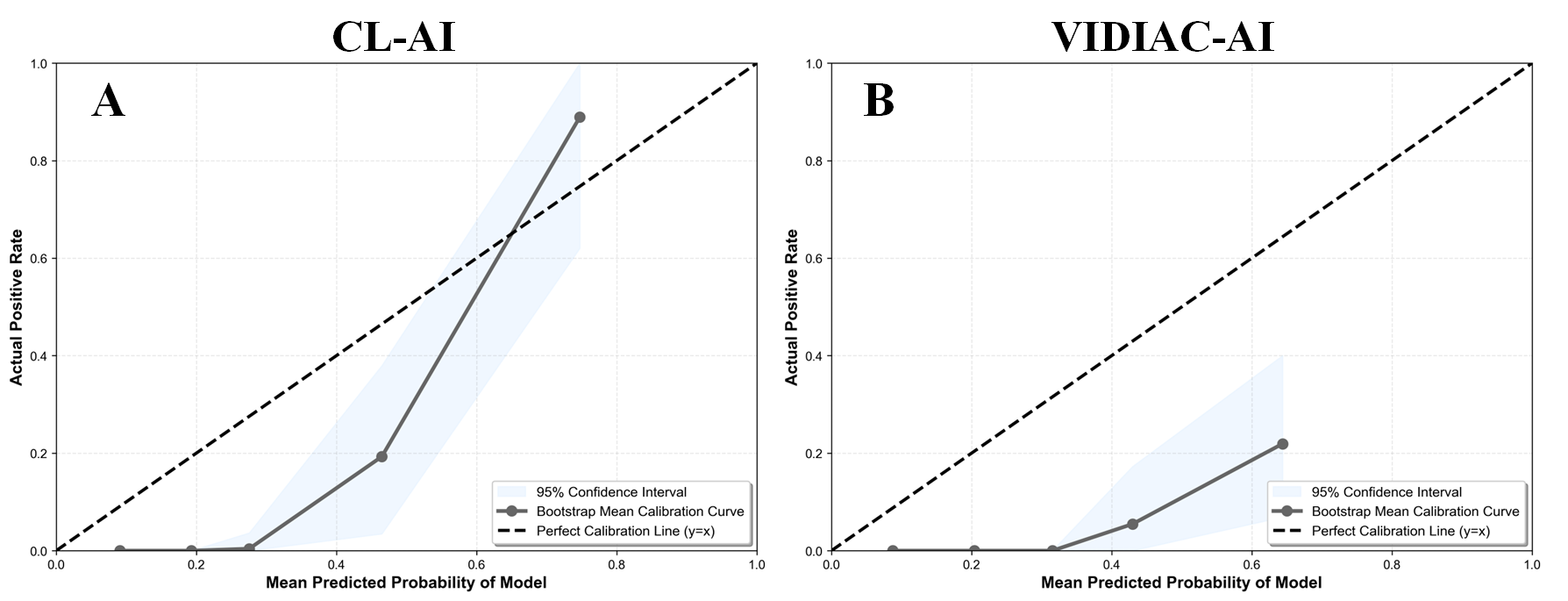

Supplement: S1 Fig — (A) Calibration plot of the CL-AI model. (B) Calibration plot of the VIDIAC-AI model. Predicted probabilities were grouped into five bins according to the mean predicted risk. The solid line with points represents the mean observed proportion of difficult airway events within each bin, and the shaded area indicates the 95% confidence interval estimated using 1,000 bootstrap resamples. The dashed diagonal line represents perfect calibration (y = x). Wider uncertainty and deviations from the ideal line, particularly for the VIDIAC-AI model, are expected given the limited number of positive cases in the test set. (TIF) [file pone.0342339.s007.tif]
